# Characterizing the Penumbras of White Matter Hyperintensities and Their Associations With Cognitive Function in Patients With Subcortical Vascular Mild Cognitive Impairment

**DOI:** 10.3389/fneur.2019.00348

**Published:** 2019-04-12

**Authors:** Xiaowei Wu, Xin Ge, Jing Du, Yao Wang, Yawen Sun, Xu Han, Weina Ding, Mengqiu Cao, Qun Xu, Yan Zhou

**Affiliations:** ^1^Department of Radiology, Ren Ji Hospital, School of Medicine, Shanghai Jiao Tong University, Shanghai, China; ^2^Department of Neurology, Ren Ji Hospital, School of Medicine, Shanghai Jiao Tong University, Shanghai, China

**Keywords:** white matter hyperintensity, normal appearing white matter, penumbra, cerebral blood flow, diffusion tensor imaging, subcortical vascular mild cognitive impairment

## Abstract

Normal-appearing white matter (NAWM) surrounding white matter hyperintensities (WMHs), frequently known as the WMH penumbra, is associated with subtle white matter injury and has a high risk for future conversion to WMHs. The goal of this study was to define WMH penumbras and to further explore whether the diffusion and perfusion parameters of these penumbras could better reflect cognitive function alterations than WMHs in subjects with subcortical vascular mild cognitive impairment (svMCI). Seventy-three svMCI subjects underwent neuropsychological assessments and 3T MRI scans, including diffusion tensor imaging (DTI) and arterial spin labeling (ASL). To determine the extent of cerebral blood flow (CBF) and DTI penumbras. A NAWM layer mask was generated for periventricular WMHs (PVWMHs) and deep WMHs (DWMHs) separately. Mean values of CBF, fractional anisotropy (FA), mean diffusivity (MD) within the WMHs and their corresponding NAWM layer masks were computed and compared using paired *t*-tests. Pearson's partial correlations were used to assess the relations of the mean CBF, FA, and MD values within the corresponding penumbras with composite z-scores of global cognition and four cognitive domains controlling for age, sex, and education. For both PVWMHs and DWMHs, the CBF penumbras were wider than the DTI penumbras. Only the mean FA value of the PVWMH-FA penumbra was correlated with the composite z-scores of global cognition before correction (*r* = 0.268, *p* = 0.024), but that correlation did not survive after correcting the *p*-value for multiple comparisons. Our findings showed extensive white matter perfusion disturbances including white matter tissue, both with and without microstructural alterations. The imaging parameters investigated, however, did not correlate to cognition.

## Introduction

Vascular cognitive impairment (VCI) refers to all levels of cognitive alteration, ranging from mild to severe, caused by cerebrovascular disease (1). Subcortical vascular cognitive impairment (SVCI) is a common form of VCI caused by subcortical ischemic vascular disease (SIVD) (2). Subcortical vascular mild cognitive impairment (svMCI) is a prodromal stage of Subcortical vascular dementia (SVaD) (3). It is clinically important to focus on svMCI before it develops into SVaD. White matter hyperintensities (WMHs), also known as WM lesions or leukoaraiosis, are usually considered the most common magnetic resonance imaging (MRI) manifestations of SIVD, increasing with both age and vascular risk factors (4, 5). WMHs are frequently divided into periventricular WMH (PVWMH) and deep WMH (DWMH), and the two classifications have been differentially associated with vascular risk factors (6–9), cognitive function measures (10–14) and histopathologic findings (14–17). The burden of WMHs has widely been reported to be associated with cognitive decline and the progression of cognitive impairment (13, 18–22).

There is an increasing amount of evidence suggesting that WM alterations are not only present in visible WMHs but also in the normal-appearing WM (NAWM) surrounding WMHs, which conventional MRI scans re unable to identify. Previous reports have indicated that NAWM displays subtle immunohistochemical and pathological alterations beyond WM lesions, raising the possibility that WM dysfunction is more widespread than the focal WM lesions (23–26). Previous studies have also demonstrated WM integrity disruption and hypoperfusion within NAWM surrounding WMHs, using diffusion tensor imaging (DTI) and arterial spin labeling (ASL) (27–31). The specific, subtly changed NAWM surrounding WMHs is called a “WMH penumbra” in many studies; however, this terminology has not yet been widely adopted.

WMH penumbras represent milder WM injury concerning WMHs and are at a higher risk for future conversion to WMH than other areas of healthy WM beyond penumbras (29). Previous studies have shown that structural WMH penumbras, defined by DTI and FLAIR intensity, extended approximately 2–9 mm, while cerebral blood flow (CBF) WMH penumbras, defined by ASL, extended ~12–14 mm around both PVWMH and DWMH in cognitively intact community-dwelling elderly individuals (27–29, 32). Though the extent of WMH penumbras is not entirely consistent among studies, these findings consistently reveal that CBF penumbras are more extensive than structural penumbras, indicating that altered CBF may predate structural changes in the NAWM surrounding WMH. Longitudinal studies have further demonstrated that WMHs and their penumbras represent a continuum of WM injury that evolves over time and that abnormal changes in surrounding NAWM precede the progression of WMH (27, 28, 32, 33). Other studies have also found that most of the new lacunes preferentially localize to WMH penumbras and that infarcts on the edge of WMHs are more likely to develop into lacunes or cavities than those far from WMHs (34, 35). These findings imply that WMH penumbras can be regarded as relevant clinical targets for interventions that prevent the development of WMHs. Structural penumbras defined by DTI may provide information about full-scale microstructural WM integrity disruption, and CBF penumbras may provide etiological insight into the formation and progression of WMHs. Therefore, it is crucial to combine structural and CBF penumbras of WMHs within svMCI patients to facilitate our understanding of the mechanism underlying WMH progression and cognitive impairment evolution.

Currently, studies characterizing both structural and CBF WMH penumbra in the same svMCI subjects are lacking. The goal of this study is to characterize the CBF and structural penumbras of PVWMHs and DWMHs and to further explore whether penumbras can reflect cognitive function alterations, better than WMHs themselves, among svMCI subjects.

## Materials and Methods

### Subjects

SIVD subjects were recruited from patients who were admitted to the Neurology Department of Ren Ji Hospital between August 2015 and December 2017. SIVD was defined as a subcortical WM hyperintensity on T2-FLAIR imaging with at least one lacunar infarct, according to the criteria suggested by Galluzzi et al. (36). SIVD subjects who fulfilled svMCI criteria, suggested by Petersen et al. (37) and Gorelick et al. (38), were included in our study. The inclusion criteria were as follows: (1) subjective cognitive difficulty reported by the patient or caregiver; (2) quantifiable cognitive decline within one or more cognitive domains (e.g., attention-executive function, memory, language, or visuospatial function); (3) normal instrumental activity of daily living. The exclusion criteria were as follows: (1) cortical and/or corticosubcortical non-lacunar territorial infarcts and watershed infarcts; (2) neurodegenerative diseases (including Parkinson's disease and AD); (3) signs of normal-pressure hydrocephalus; (4) specific causes of WM lesions (e.g., metabolic, toxic, infectious, multiple sclerosis, brain irradiation); (5) alcoholic encephalopathy or illicit drug use; (6) major depression (Hamilton Depression Rating Scale (HDRS) ≥ 18) (39); (7) severe cognitive impairment (inability to perform the neuropsychological test or undergo the whole MRI scan); (8) MRI safety contraindications and claustrophobia; (9) education ≤ 6 years. Early VCI is characterized by executive function/processing speed deficits with relatively preserved memory and is less likely to produce subjective complaints, whereas Alzheimer's disease (AD) or mixed cognitive impairment feature memory problems (2). Thus, we carefully excluded the participants with memory complaints. All patients underwent laboratory examinations to exclude systemic or other neurological diseases. Finally, 73 right-handed svMCI patients were included in this study.

The present research was approved by the Research Ethics Committee of the Ren Ji Hospital, School of Medicine, Shanghai Jiao Tong University. Written informed consent was obtained from each subject before participation. All procedures were in accordance with the institutional guidelines.

### Neuropsychological Assessment

Neuropsychological assessments were performed by two experienced neurologists (QX and WC) within 1 week of the MRI examination. No patients suffered any transient ischemic attacks or strokes between the MRI examination and the assessment. A comprehensive battery of neuropsychological tests was designed to evaluate cognitive status, which included all cognitive domains. The scales listed in Table 1 were used as described in previous studies (40, 41). To assess the cognitive status of each patient, the norms used were based on the mean score of each measurement, which were derived from a small-scale normative study of a community of healthy elderly people in Shanghai, China (42). Cognitive dysfunction was defined as −1.5 SD on at least one neuropsychological test.

**Table 1:** Neuropsychological tests used to evaluate cognitive status.

Attention/executive function	Chinese modified version of the Trail Making Test (TMT) Modified version of the Stroop Color-Word Test (SCWT) Category Verbal Fluency Test (VFT)
Memory	Chinese version of the Auditory Verbal Learning Test (AVLT) for short-delay and long-delay free recall Rey-Osterrieth Complex Figure (ROCF) delayed recall test (Chinese version)
Language	Boston Naming Test (the 30-item version)
Visuospatial function	ROCF copy test

To allow direct comparisons among different neuropsychological tests, a z-score was calculated for each neuropsychological test. A z-score is defined as a score that falls in the distribution of normal scores. A z-score of +1.0 corresponds to a score 1 SD above the mean score. The raw scores for each neuropsychological measure were z-transformed. Then, the z-scores for each domain were generated by averaging the z-scores of their respective tests. Composite z-scores of global cognition, which represented general intellectual ability, were computed by averaging the z-scores of all four cognitive domains.

### MRI Acquisition

All MRI data were obtained using a 3.0T MRI scanner (Signa HDxt; GE HealthCare, Milwaukee, WI, USA) equipped with an eight-channel phased array head coil at Ren Ji Hospital. Each subject underwent three dimensional T1 high-resolution imaging and FLAIR scans. All subjects underwent MR diffusion tensor imaging (DTI) scans. Fifty-six of the seventy three subjects underwent three-dimensional arterial spin labeling (3DASL) scans. The parameters of each sequence were as follows: (1) sagittal 3D T1 high-resolution imaging [repetition time (TR) = 5.6 ms, echo time (TE) = 1.8 ms, inversion time (TI) = 450 ms, flip angle = 15°, slice thickness = 1.0 mm, number of slices = 156, gap = 0, field of view (FOV) = 256 mm × 256 mm, and matrix = 256 × 256]; (2) axial FLAIR (TR = 9,075 ms, TE = 150 ms, TI = 2,250 ms, FOV = 256 mm × 256 mm, matrix = 256 × 256, slice thickness = 2 mm, and number of slices = 66); (3) 3D ASL perfusion images were acquired using 3D fast spin-echo acquisition with background suppression and with a labeling duration of 1,500 ms and a post labeling delay of 2,000 ms. (TR = 4,337 ms, TE = 9.8 ms, FOV = 240 mm × 240 mm, slice thickness = 4 mm, flip angle = 155°, NEX = 3, and number of slices = 34); (4) DTI (TR = 17,000 ms, TE = 89.8 ms, slice thickness = 2 mm, gap = 0, FOV = 256 mm × 256 mm, number of slices = 66, matrix = 128 × 128, and 20 diffusion-weighted directions with b value = 1,000 s/mm^2^).

### Image Processing and Analysis

Processing of the diffusion MRI dataset was implemented using a pipeline toolbox, PANDA v1.3.1(https://www.nitrc.org/projects/panda), which is based on FSL tools (43). In the pipeline, skull-stripping with the brain extraction tool (BET) was done to extract brain tissue for b0 image in each subject. Eddy current-induced distortion and head motion artifacts were corrected by registering each raw diffusion-weighted image to the b0 image with an affine transformation. Then diffusion metrics, including FA and MD, were calculated within a mask created from b0 image. To derive the CBF map of each subject, the three runs of ASL images were inspected, and data with excessive head movement (≥2 mm or 2°) were discarded, and the three runs were concatenated. Quantitative CBF was then calculated on a voxel basis according to Wang et al. (44). Voxel-wise partial volume correction was performed (45). Then, the CBF, FA and MD maps for each subject were coregistered to the corresponding individual 3D T1-weighted images using SPM8 (http://www.fil.ion.ucl.ac.uk/spm/software/spm8/).

WMHs were segmented by the lesion growth algorithm as implemented in the LST toolbox version 1.1.4 (www.statistical-modeling.de/lst.html) for SPM (46). Then, WMH clusters were separated into PVWMHs and DWMHs according to the “continuity to ventricle rule” (47). PVWMHs were defined as WMHs that were continuous with the margin of the lateral ventricle and all others were defined as DWMHs. Finally, probabilistic maps for WMH and WM were processed with binarization.

**Figure 1:**
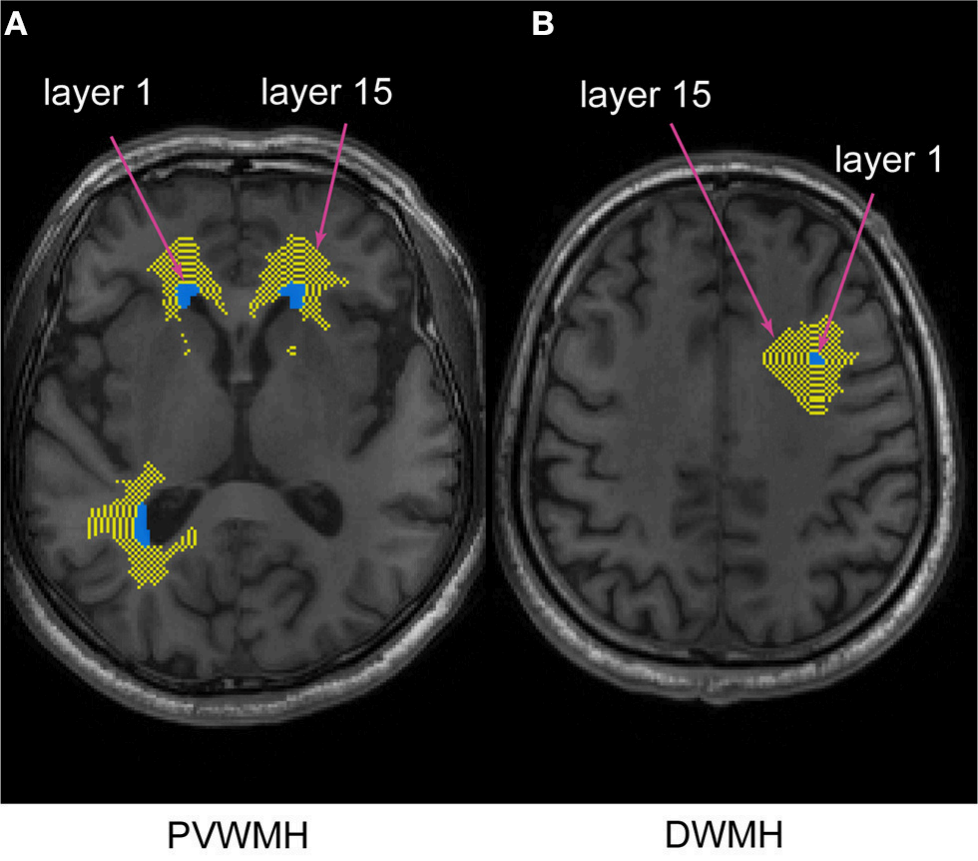
The spatial relationship between PVWMH, DWMH, and their corresponding NAWM layer masks. The turquoise and yellow represent WM lesions and NAWM layers, respectively. The innermost layer surrounding the WMH represents layer 1, and the outermost layer represents layer 15. **(A)** periventricular white matter hyperintensity (PVWMH); **(B)** deep white matter hyperintensity (DWMH).

To assess the WMH penumbra for each imaging measure, a NAWM layer mask for each individual dataset was created by linearly aligning the defined binary WMHs to the T1-weighted images, according to previous studies (27, 28). The NAWM layer mask consisted of 15 layers of PVWMH and DWMH separately. Each layer was parallel and gradually dilated away from the WMH by 1 mm. The innermost layer, closest to the WMH, was defined as layer 1 (NAWM-L1), and the outermost layer was layer 15 (NAWM-L15). To prevent overlapping between layers of neighboring WMHs, the WMH and previous NAWM layers were merged together to create a new “WMH” before creating the next layer. To reduce the partial volume effects of the gray matter (GM) and cerebrospinal fluid (CSF), the GM and CSF maps were dilated by 2 voxels, and subtracted from the NAWM layers. The spatial relationship between WMH and NAWM layer mask is shown in Figure 1.

For each subject, the NAWM layer mask was applied to the FA, MD, and CBF maps which were previously coregistered to individual 3D T1-weighted images. Later, the mean FA, MD, and CBF values of each NAWM layer for the PVWMH and the DWMH were computed. Similarly, the imaging parameter values of whole-brain WMH and its subtype and whole-brain NAWM were obtained.

### Statistical Analysis

The analyses were performed using IBM SPSS Statistics 20 and R version 3.5.0. To determine the extent of WMH CBF penumbra, mean CBF of the WMH and NAWM layers (L1–L15) were compared with the corresponding mean CBF of whole-brain NAWM using a paired *t*-test. The first layer whose CBF value was not significantly different from the whole-brain NAWM was defined as the outer boundary of the CBF penumbra. Due to the impact of WM association and commissural fibers surrounding the ventricles on DTI parameters, the extent of WMH structural penumbra was determined by comparing every two adjacent NAWM layers using paired *t*-tests. The first of two neighboring layers whose values were not significantly different from each other was defined as the outer boundary of the structural penumbra. The PVWMH and DWMH were analyzed separately. Pearson's partial correlations were used to assess the relation between the mean CBF, FA, and MD values of PVWMH, DWMH, and their corresponding penumbras with composite z-scores, controlling for age, sex, and education. A significant difference was set at *p* ≤ 0.05.

## Results

### Participant Characteristics

The demographic and cognitive characteristics were presented in Table 2. Seventy-three subjects were included in our study. Their age ranged from 50 to 86 years, with the mean years of education 10.47 ± 3.07. The composite z-scores of global cognition were -0.86 ± 0.87, and the z-scores of four cognitive domain (attention/executive function, memory, language and visuospatial function) were -1.45 ± 1.26, 1.46 ± 0.83, -0.75 ± 1.56 and 0.22 ± 1.99, respectively.

**Table 2:** Summary of participant characteristics.

**Variables**	**Mean ± SD (range)**
Number of subjects	73
Age (years)	65.71 ± 8.2 (~50–86)
Female	25
Years of education	10.47 ± 3.07 (~6–18)
Z-scores of attention/executive function	−1.45 ± 1.26 (~–5.33–1.12)
Z-scores of memory	−1.46 ± 0.83 (~–3.05–0.48)
Z-scores of language	−0.75 ± 1.56 (~–5.05–1.79)
Z-scores of visuospatial function	0.22 ± 1.99 (~–8.19–1.46)
Composite z-scores of global cognition	−0.86 ± 0.87 (~–4.04–0.66)

*SD, standard deviation*.

### The Mean FA, MD, and CBF Values of Each NAWM Layer of the PVWMH and DWMH Are Presented in Table 3 and Figure 2

The extents of the PVWMH penumbras are as follows: 10 mm for the CBF penumbra; 6 mm for the FA penumbra; 6 mm for the MD penumbra. The extents of the DWMH penumbras are as follows: 7 mm for the CBF penumbra; 4 mm for the FA penumbra; 2 mm for the MD penumbra (see Tables S1–S3).

**Table 3:** The mean FA, MD, and CBF values of the PVWMH, DWMH, and their corresponding layers (mean ± SD).

	**CBF(ml/100 g-tissue/min)**		**FA**		**MD(10**^****−4****^**mm**^****2****^**/s)**	
	**PVWMH**	**DWMH**	**PVWMH**	**DWMH**	**PVWMH**	**DWMH**
WMH	22.20 ± 6.85	30.72 ± 9.74	0.266 ± 0.031	0.289 ± 0.057	13.62 ± 1.52	10.34 ± 1.77
Layer 1	23.66 ± 7.05	29.94 ± 8.83	0.310 ± 0.041	0.310 ± 0.056	11.48 ± 1.46	9.86 ± 1.60
Layer 2	24.73 ± 7.11	30.58 ± 8.67	0.322 ± 0.048	0.316 ± 0.056	10.99 ± 1.56	9.69 ± 1.53
Layer 3	25.85 ± 7.22	30.93 ± 8.62	0.329 ± 0.052	0.321 ± 0.056	10.75 ± 1.52	9.68 ± 1.59
Layer 4	26.97 ± 7.29	31.31 ± 8.67	0.334 ± 0.053	0.324 ± 0.054	10.51 ± 1.46	9.63 ± 1.63
Layer 5	28.06 ± 7.39	31.68 ± 8.76	0.337 ± 0.055	0.325 ± 0.052	10.37 ± 1.50	9.75 ± 1.67
Layer 6	29.11 ± 7.53	32.18 ± 8.91	0.339 ± 0.056	0.325 ± 0.050	10.18 ± 1.43	9.72 ± 1.67
Layer 7	30.02 ± 7.62	32.72 ± 9.05	0.338 ± 0.056	0.325 ± 0.048	10.11 ± 1.36	9.77 ± 1.74
Layer 8	31.22 ± 7.72	33.18 ± 9.14	0.338 ± 0.056	0.325 ± 0.048	10.03 ± 1.33	9.75 ± 1.69
Layer 9	32.25 ± 7.85	33.72 ± 9.26	0.336 ± 0.055	0.325 ± 0.046	10.03 ± 1.30	9.73 ± 1.72
Layer 10	33.17 ± 7.97	34.10 ± 9.28	0.332 ± 0.055	0.324 ± 0.045	10.01 ± 1.29	9.75 ± 1.73
Layer 11	33.82 ± 8.37	34.40 ± 9.19	0.328 ± 0.055	0.323 ± 0.044	10.00 ± 1.26	9.83 ± 1.69
Layer 12	34.67 ± 8.13	34.88 ± 9.20	0.324 ± 0.054	0.321 ± 0.043	9.99 ± 1.27	9.83 ± 1.60
Layer 13	35.28 ± 8.14	35.26 ± 9.24	0.320 ± 0.054	0.319 ± 0.042	9.96 ± 1.31	9.92 ± 1.61
Layer 14	35.77 ± 8.18	35.52 ± 9.18	0.315 ± 0.054	0.317 ± 0.042	9.97 ± 1.32	9.94 ± 1.60
Layer 15	36.21 ± 8.30	35.76 ± 8.93	0.311 ± 0.056	0.315 ± 0.042	10.01 ± 1.37	9.97 ± 1.53
WBNAWM	33.45 ± 8.58	33.45 ± 8.58	0.312 ± 0.026	0.312 ± 0.026	9.19 ± 0.74	9.19 ± 0.74

*FA, fractional anisotropy; MD, mean diffusivity; CBF, cerebral blood flow; WMH, white matter hyperintensity; WBNAWM, whole brain normal-appearing white matter; SD, standard deviation*.

**Figure 2:**
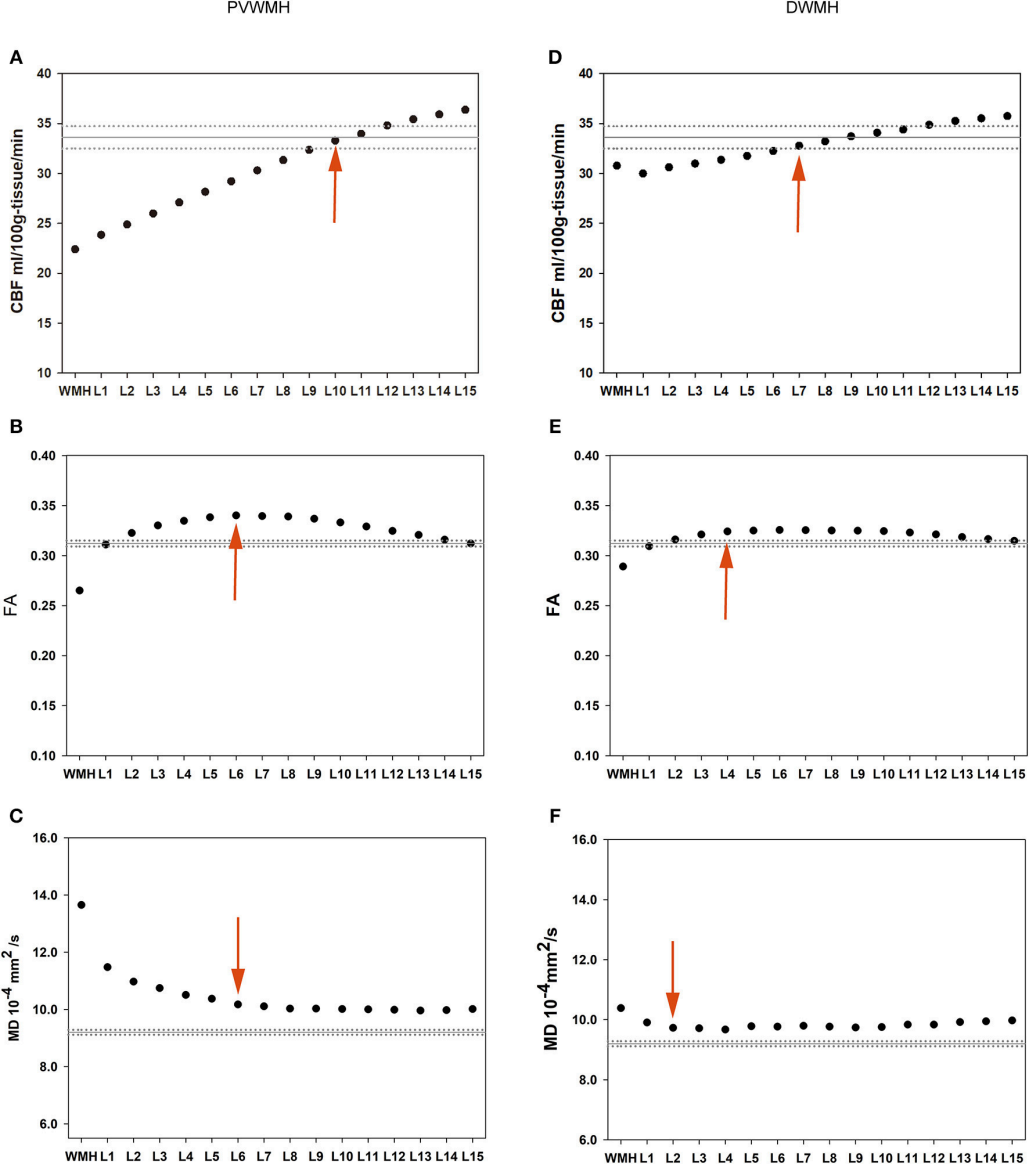
Group means of the PVWMH (the left column) and DWMH (the right column) and their outer NAWM layers. The solid horizontal and dotted lines represent the mean and standard error of the whole-brain NAWM CBF, FA, and MD values, respectively. Red arrows represent the outer boundary of WMH penumbra for each dataset. **(A)** PVWMH-CBF; **(B)** PVWMH-FA; **(C)** PVWMH-MD; **(D)** DWMH-CBF; **(E)** DWMH-FA; **(F)** DWMH-MD.

### Relations Between the Mean FA, MD, and CBF Values of the PVWMH, DWMH, and Their Penumbras With Composite z-Scores of Global Cognition and the z-Scores of Each Cognitive Domain Are Illustrated in Table 4 and Tables S4–S7

**Figure 3:**
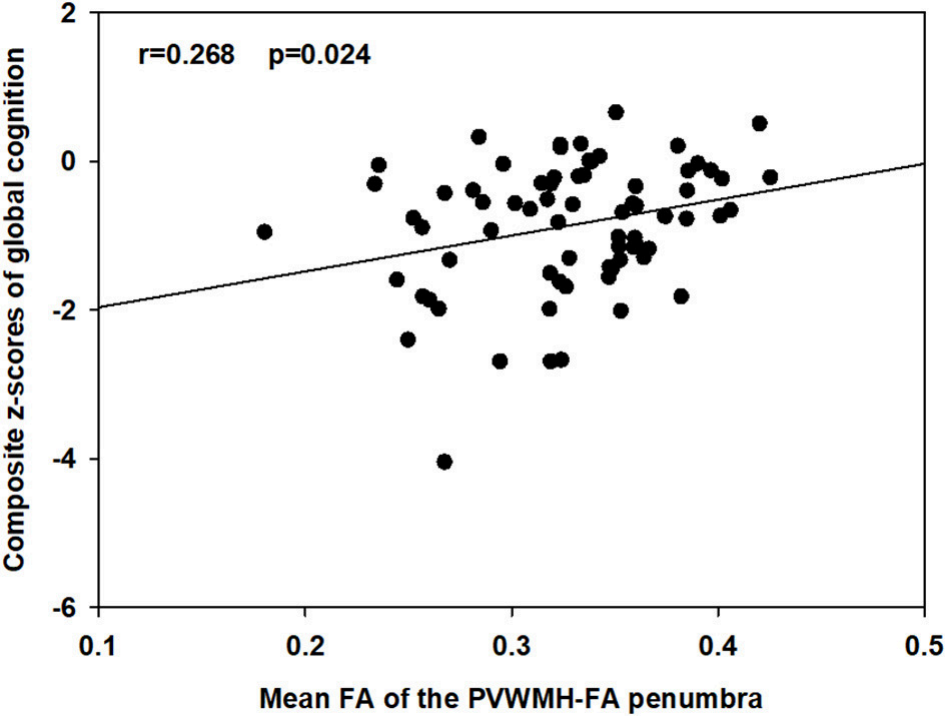
Associations between the mean FA of the PVWMH-FA penumbra and the composite z-scores of global cognition. Partial *r* and *p*-values was obtained after controlling for age, sex, and education.

Only the mean FA value of the PVWMH-FA penumbra was correlated with the composite z-scores of global cognition (*r* = 0.268, *p* = 0.024) before correction, as showed in Figure 3. After false discovery rate correction, that correlation did not survive.

**Table 4:** Relations between the mean FA, MD, and CBF values of PVWMH, DWMH, and their penumbras with composite z-scores of global cognition.

	**Mean values of imaging parameters**	***r***	***p*-value**	***p*-value corrected by FDR**
PVWMH-CBF	22.20	0.112	0.421	0.6495
PVWMH-FA	0.267	0.115	0.341	0.6495
PVWMH-MD	13.62	0.077	0.522	0.6960
DWMH-CBF	30.72	0.171	0.226	0.6945
DWMH-FA	0.289	0.046	0.707	0.7370
DWMH-MD	10.34	−0.13	0.286	0.6945
Mean CBF of PVWMH-CBF penumbra	28.52	−0.047	0.737	0.7370
Mean FA of PVWMH-FA penumbra	0.328	0.268	0.024	0.2880
Mean MD of PVWMH-MD penumbra	10.71	−0.047	0.696	0.7370
Mean CBF of DWMH-CBF penumbra	31.33	0.213	0.129	0.6945
Mean FA of DWMH-FA penumbra	0.318	0.096	0.433	0.6945
Mean MD of DWMH-MD penumbra	9.8	−0.110	0.370	0.6945

*The p-values are corrected by false discovery rate later on. PVWMH, periventricular white matter hyperintensity; DWMH, deep white matter hyperintensity; CBF, cerebral blood flow (ml/100 g-tissue/min); FA, fractional anisotropy; MD, mean diffusivity (10^-4^mm^2^/s); FDR, false discovery rate*.

## Discussion

For SVCI subjects and cognitively intact elder individuals, the morphological characteristics and distribution features of WM lesions have been widely reported to be associated with cognitive decline (12, 13, 48, 49), whereas few studies have been conducted to explore the association between WMH penumbras and cognitive function and the contribution of subtle abnormalities within the penumbras to cognitive function remains unclear. The present study aimed to explore the role of WMH penumbras in global cognition of svMCI subjects. Our results revealed reduced CBF and FA, and increased MD, of WMH and its surrounding NAWM for both PVWMH and DWMH. Our findings also showed that the CBF penumbra was wider than the structural penumbra as defined by FA and MD.

Our finding that CBF penumbras were more extensive than structural penumbras around both PVWMH and DWMH was consistent with a previous study (27). Wider CBF penumbras covered structural penumbras and no microstructural changed NAWM, suggesting the likelihood that compromised CBF precedes white matter integrity changes. Longitudinal research is needed to determine whether perfusion or structural changes come first. CBF plays a critical role in the maintenance of neuronal integrity, and the CBF penumbra may reflect more extensive white matter alterations beyond the structural penumbra. A previous longitudinal study found that NAWM voxels that converted to new WMH at follow-up had significantly lower baseline CBF than persistent NAWM voxels that did not convert to WMH, suggesting that the CBF penumbra is linked to WMH extension (28). Lower CBF within NAWM reflects a higher risk for future conversion to WMH. Thus, the CBF penumbras of both PVWMH and DWMH can be considered novel targets for prediction and intervention of WMH progression. Our findings showed that CBF gradually increased from NAMW layer 1 to layer 15, suggesting that subtle NAWM injury is likely a continuous process, and WMH growth is mostly from inner NAWM layers of existing WMHs to outer layers. This finding supported those of previous studies, revealing that most growing WMHs extended from the edge of existing lesions to the outer region (22, 28).

Consistent with previous studies, we found that the FA and MD penumbras covered 2–6 mm from WMHs (22, 27, 29). Our results showed that the FA values of the inner periventricular NAWM layers were slightly higher than those of the outer periventricular NAWM layers and the mean whole-brain NAWM. The “location effect” may explain this phenomenon (27). PVWMHs are located near highly organized myelinated structures, such as the corpus callosum, where water diffusion is highly restricted and dependent on fiber direction. Other studies have also shown similar phenomena (27, 50). DTI measures of WM microstructural integrity appeared to provide an earlier indication of WM injury than WMHs. A previous study demonstrated that NAWM regions converting to WMH had significantly lower FA and higher MD values than persistent NAWM regions, suggesting that changes in NAWM precede the development of WM lesions (33). Moreover, vast number of studies have revealed that the pathophysiological alterations in WM is a gradual process and WMHs are only the tip of the iceberg of WM pathology (23, 29, 51, 52). Decreased FA and increased MD within NAWM and WMH indicate microstructural WM integrity disruption and altered water mobility, which may affect the integrity of WM tracts connecting cortical-subcortical areas, leading to disconnection syndrome and cognitive decline (53, 54).

Only the PVWMH-FA penumbra was correlated with global cognition, whereas that correlation did not survive after correcting the *p*-value for multiple comparisons. The relationship between penumbra findings and cognition was weak. This likely represents the fact that these physiological processes of penumbra are no destructive per se but are established risk factors for future brain injury that will associated with progressive cognitive impairment. However, the exact reason underlying this occurrence is not well-established, and further studies are needed.

Our finding that there was no significant correlation between the imaging parameters within WMHs, including the two classifications, and global cognitive function was not consistent with those of previous studies (55, 56). A previous study revealed that global cognitive function, assessed by Mini-Mental State Examination (MMSE), was associated with total brain NAWM-FA and WMH-FA in subjects with leukoaraiosis using a multiple linear regression analysis (55). A population-based study also showed that MD, radial diffusivity and axial diffusivity of both WMH and NAWM were associated with global cognition, regardless of white matter atrophy and WMH volume (57). The lack of correlation between imaging parameters and cognition in our study, which was inconsistent with these previous studies, may be attributed to two aspects as follows. On the one hand, this discrepancy may be attributed to the different participants and methods used to assess cognitive status. On the other hand, the heterogeneity of WMHs may partly explain this discrepancy. Postmortem studies have revealed that WMHs are histopathologically heterogeneous in both severity and nature (26). The spatial distribution and the signal properties, including magnetization transfer imaging, DTI and FLAIR, of WMHs have also been revealed to be heterogeneous, though WMHs have similar appearances on FLAIR and T2-weighted images (32, 58, 59). Given that WMHs are heterogeneous in terms of histopathology, spatial distribution and the signal properties, mixed analysis of WMHs may compromise the reliability of the analysis in assessing the association of WMHs with cognitive function, which may partly explain our finding. Though it is not feasible or realistic to control all the heterogeneities of WMHs in one study, future studies should be conducted to stratify WMHs according to their heterogeneities.

Measures of microstructural integrity and perfusion within WMH penumbra may have several clinical implications. First, to prevent WMH growth, it is crucial to understand the etiology of development of NAWM tissue within WMH penumbra into WMH which represents more severe WM injury. A longitudinal study had showed that radial diffusivity, reflecting demyelination (60, 61), had the strongest relationship with WMH expansion compared to axial diffusivity and CBF, indicating that demyelination may be the main underlying etiology of WMH development (62). Second, previous longitudinal studies had found that some WMH may regress after minor stroke, with potentially better clinical and brain tissue outcomes, suggesting that WMH reversibility may attribute to the transient disturbance of the blood-brain barrier (BBB), causing interstitial fluid alterations (63–66). A longitudinal study revealed that participants with WMH decrease had larger reductions in blood pressure and MD in NAWM than participants with WMH increase (66). Thus, ASL CBF and DTI measures may be used to investigate cerebral perfusion and microstructural integrity as the potential mechanism explaining the reversal of WMH and WMH penumbra. Third, a clinician should take into account that the true WM injury may be more extensive than the visible WMH. WMH penumbra represents milder WM injury concerning WMH and may be a high-priority target for intervention as it is potentially reversible to treatment.

There were several limitations to our study. First, because there is no generally adopted rule for defining the extent of WMH penumbras to date, we defined the CBF penumbra by comparing the mean CBF of each NAWM layer to that of the whole-brain NAWM. Whole-brain NAWM already contained subtle changes in the NAWM. Thus, the CBF penumbra defined by this method is slightly narrower than the real CBF penumbra. Second, the number of lacunes, WMH location and corresponding cortical dysfunction also have an impact on cognitive impairments (67, 68). The associations between WMHs and their penumbras with cognition may be mediated by these confounders. In the present study, we only investigated the effect of WMH penumbras on cognitive status. Our future work will seek to quantify and model these confounders for the purpose of better understanding the role of WMH penumbra in cognition. Third, in this study, we assumed that WMHs equably affects surrounding NAMW along the straight line between the two, without taking the architecture of WM fibers into consideration. It may be more biologically plausible to assume that a WMH at one location on a WM fiber more strongly influences WM integrity along the rest of the same tract than in other tracts. However, it seems to be unfeasible and unrealistic to use DTI tractography to investigate each WMH and its penumbra one by one. Finally, our study was a cross-sectional study, and the sample size was small. Longitudinal studies with larger sample sizes are needed.

## Conclusion

In this study, reduced CBF and FA and increased MD in the inner NAWM layers for both PVWMH and DWMH suggested extensive WM alterations beyond the visible WM lesions commonly observed on clinical MRI of svMCI subjects. CBF penumbras cover more extensive WM at risk than DTI penumbras, suggesting the likelihood that compromised CBF precedes white matter integrity changes, and CBF penumbras may be a potential target for the prevention of further microstructural white matter damage. The imaging parameters investigated, however, did not correlate to cognition.

## Author Contributions

YZ, QX, and XG conceived and designed the experiments. XW, XH, JD,YS, WD, and MC performed the experiments. XW, XG, and YW analyzed the data. XW, XG, and JD contributed reagents, materials, analysis tools. XW and YW wrote the paper. XW and JD figures processing.

### Conflict of Interest Statement

The authors declare that the research was conducted in the absence of any commercial or financial relationships that could be construed as a potential conflict of interest.
