# Supplementary material for: Characterizing the Penumbras of White Matter Hyperintensities and Their Associations With Cognitive Function in Patients With Subcortical Vascular Mild Cognitive Impairment
Source: Front Neurol. 2019 Apr 12;10:348. doi: 10.3389/fneur.2019.00348 (PMC6474292; doi:10.3389/fneur.2019.00348)
Supplement: Supplementary file 1 [file Data_Sheet_1.docx]

**Table S1:** A comparison of the CBF of PVWMH and DWMH and their NAWM layers( L1-L15) with mean whole brain NAWM CBF using paired t-tests.

|  | p-values of PVWMH CBF and its NAWM layers vs whole brain NAWM CBF | p-values of DWMH CBF and its NAWM layers vs whole brain NAWM CBF |
| --- | --- | --- |
| WMH | p＜0.001 | p= 0.002814 |
| Layer 1 | p＜0.001 | p＜0.001 |
| Layer 2 | p＜0.001 | p＜0.001 |
| Layer 3 | p＜0.001 | p＜0.001 |
| Layer 4 | p＜0.001 | p= 0.001796 |
| Layer 5 | p＜0.001 | p= 0.006953 |
| Layer 6 | p＜0.001 | p= 0.041338 |
| Layer 7 | p＜0.001 | p= 0.206432 |
| Layer 8 | p＜0.001 | p= 0.555272 |
| Layer 9 | p= 0.016386 | p= 0.845735 |
| Layer 10 | p= 0.555309 | p= 0.444423 |
| Layer 11 | p= 0.425573 | p= 0.222968 |
| Layer 12 | p= 0.005254 | p= 0.060750 |
| Layer 13 | p＜0.001 | p= 0.019117 |
| Layer 14 | p＜0.001 | p= 0.006178 |
| Layer 15 | p＜0.001 | p= 0.001574 |

NAWM=normal appearing white matter; PVWMH=periventricular white matter hyperintensity; DWMH=deep white matter hyperintensity; CBF=cerebral blood flow.

Highlight represent the outer boundary of penumbra.

**Table S2:** A comparison of the FA of each two neighboring NAWM layers using paired t-tests.

|  | PVWMH NAWM layers | DWMH NAWM layers |
| --- | --- | --- |
| Layer 1 vs Layer 2 | p＜0.001 | p＜0.001 |
| Layer 2 vs Layer 3 | p＜0.001 | p＜0.001 |
| Layer 3 vs Layer 4 | p＜0.001 | p= 0.016820 |
| Layer 4 vs Layer 5 | p＜0.001 | p= 0.544308 |
| Layer 5 vs Layer 6 | p= 0.007594 | p= 0.492818 |
| Layer 6 vs Layer 7 | p= 0.373632 | p= 0.772262 |
| Layer 7 vs Layer 8 | p =0.702897 | p= 0.638103 |
| Layer 8 vs Layer 9 | p＜0.001 | p= 0.843805 |
| Layer 9 vs Layer 10 | p＜0.001 | p= 0.745763 |
| Layer 10 vs Layer 11 | p＜0.001 | p= 0.058167 |
| Layer 11 vs Layer 12 | p＜0.001 | p= 0.005397 |
| Layer 12 vs Layer 13 | p＜0.001 | p＜0.001 |
| Layer 13 vs Layer 14 | p＜0.001 | p＜0.001 |
| Layer 14 vs Layer 15 | p＜0.001 | p＜0.001 |

NAWM=normal appearing white matter; PVWMH=periventricular white matter hyperintensity; DWMH=deep white matter hyperintensity; FA=fractional anisotropy.

Highlight represent the outer boundary of penumbra.

**Table S3:** A comparison of the MD of each two neighboring NAWM layers using paired t-tests.

|  | PVWMH NAWM layers | DWMH NAWM layers |
| --- | --- | --- |
| Layer 1 vs Layer 2 | p＜0.001 | p =0.003944 |
| Layer 2 vs Layer 3 | p＜0.001 | p= 0.783714 |
| Layer 3 vs Layer 4 | p＜0.001 | p= 0.470763 |
| Layer 4 vs Layer 5 | p= 0.006658 | p= 0.058804 |
| Layer 5 vs Layer 6 | p＜0.001 | p= 0.483400 |
| Layer 6 vs Layer 7 | p= 0.058244 | p= 0.158767 |
| Layer 7 vs Layer 8 | p = 0.013270 | p= 0.620506 |
| Layer 8 vs Layer 9 | p =1.000000 | p= 0.741455 |
| Layer 9 vs Layer 10 | p= 0.320660 | p= 0.765391 |
| Layer 10 vs Layer 11 | p= 0.657835 | p= 0.013240 |
| Layer 11 vs Layer 12 | p= 0.567288 | p= 1.000000 |
| Layer 12 vs Layer 13 | p =0.158726 | p= 0.057205 |
| Layer 13 vs Layer 14 | p= 0.741384 | p= 0.418087 |
| Layer 14 vs Layer 15 | p= 0.181514 | p= 0.567390 |

NAWM=normal appearing white matter; PVWMH=periventricular white matter hyperintensity; DWMH=deep white matter hyperintensity; MD=mean diffusivity.

Highlight represent the outer boundary of penumbra.

**Table S4:** Relations between the mean FA, MD and CBF values of PVWMH,DWMH and their penumbras with z-scores of attention/executive function. The p-values are corrected by false discovery rate later on.

|  | r | p-value | p-value corrected by FDR |
| --- | --- | --- | --- |
| PVWMH-CBF | 0.051 | 0.712 | 0.9466 |
| PVWMH-FA | 0.037 | 0.762 | 0.9466 |
| PVWMH-MD | -0.020 | 0.872 | 0.9466 |
| DWMH-CBF | -0.063 | 0.659 | 0.9466 |
| DWMH-FA | -0.119 | 0.331 | 0.9466 |
| DWMH-MD | -0.008 | 0.947 | 0.9466 |
| Mean CBF of PVWMH-CBF penumbra | -0.080 | 0.563 | 0.9466 |
| Mean FA of PVWMH-FA penumbra | 0.543 | 0.186 | 0.9466 |
| Mean MD of PVWMH-MD penumbra | -0.027 | 0.823 | 0.9466 |
| Mean CBF of DWMH-CBF penumbra | -0.050 | 0.726 | 0.9466 |
| Mean FA of DWMH-FA penumbra | -0.035 | 0.777 | 0.9466 |
| Mean MD of DWMH-MD penumbra | -0.041 | 0.741 | 0.9466 |

PVWMH=periventricular white matter hyperintensity; DWMH=deep white matter hyperintensity; CBF=cerebral blood flow; FA=fractional anisotropy; MD=mean diffusivity; FDR= false discovery rate

**Table S5:** Relations between the mean FA, MD and CBF values of PVWMH,DWMH and their penumbras with z-scores of memory. The p-values are corrected by false discovery rate later on.

|  | r | p-value | p-value corrected by FDR |
| --- | --- | --- | --- |
| PVWMH-CBF | 0.033 | 0.811 | 0.8107 |
| PVWMH-FA | -0.046 | 0.703 | 0.7674 |
| PVWMH-MD | 0.068 | 0.579 | 0.7218 |
| DWMH-CBF | 0.148 | 0.294 | 0.7063 |
| DWMH-FA | -0.216 | 0.075 | 0.5864 |
| DWMH-MD | 0.131 | 0.283 | 0.7063 |
| Mean CBF of PVWMH-CBF penumbra | 0.073 | 0.602 | 0.7218 |
| Mean FA of PVWMH-FA penumbra | -0.076 | 0.529 | 0.7218 |
| Mean MD of PVWMH-MD penumbra | 0.087 | 0.479 | 0.7218 |
| Mean CBF of DWMH-CBF penumbra | 0.090 | 0.524 | 0.7218 |
| Mean FA of DWMH-FA penumbra | -0.167 | 0.171 | 0.6839 |
| Mean MD of DWMH-MD penumbra | 0.201 | 0.098 | 0.5864 |

PVWMH=periventricular white matter hyperintensity; DWMH=deep white matter hyperintensity; CBF=cerebral blood flow; FA=fractional anisotropy; MD=mean diffusivity; FDR= false discovery rate.

**Table S6:** Relations between the mean FA, MD and CBF values of PVWMH,DWMH and their penumbras with z-scores of visuospatial function. The p-values are corrected by false discovery rate later on.

|  | r | p-value | p-value corrected by FDR |
| --- | --- | --- | --- |
| PVWMH-CBF | -0.009 | 0.950 | 0.9989 |
| PVWMH-FA | 0.205 | 0.087 | 0.1877 |
| PVWMH-MD | -0.011 | 0.928 | 0.9989 |
| DWMH-CBF | 0.000 | 0.999 | 0.9989 |
| DWMH-FA | 0.284 | 0.018 | 0.1084 |
| DWMH-MD | -0.246 | 0.042 | 0.1356 |
| Mean CBF of PVWMH-CBF penumbra | -0.164 | 0.235 | 0.4030 |
| Mean FA of PVWMH-FA penumbra | 0.312 | 0.008 | 0.0982 |
| Mean MD of PVWMH-MD penumbra | -0.115 | 0.345 | 0.5178 |
| Mean CBF of DWMH-CBF penumbra | 0.001 | 0.992 | 0.9989 |
| Mean FA of DWMH-FA penumbra | 0.242 | 0.045 | 0.1356 |
| Mean MD of DWMH-MD penumbra | -0.203 | 0.094 | 0.1877 |

PVWMH=periventricular white matter hyperintensity; DWMH=deep white matter hyperintensity; CBF=cerebral blood flow; FA=fractional anisotropy; MD=mean diffusivity; FDR= false discovery rate.

**Table S7:** Relations between the mean FA, MD and CBF values of PVWMH,DWMH and their penumbras with z-scores of language. The p-values are corrected by false discovery rate later on.

|  | r | p-value | p-value corrected by FDR |
| --- | --- | --- | --- |
| PVWMH-CBF | 0.075 | 0.592 | 0.9093 |
| PVWMH-FA | 0.009 | 0.940 | 0.9395 |
| PVWMH-MD | 0.156 | 0.199 | 0.7978 |
| DWMH-CBF | 0.195 | 0.165 | 0.7978 |
| DWMH-FA | -0.075 | 0.543 | 0.9093 |
| DWMH-MD | 0.012 | 0.922 | 0.9395 |
| Mean CBF of PVWMH-CBF penumbra | -0.072 | 0.606 | 0.9093 |
| Mean FA of PVWMH-FA penumbra | 0.076 | 0.529 | 0.9093 |
| Mean MD of PVWMH-MD penumbra | 0.089 | 0.466 | 0.9093 |
| Mean CBF of DWMH-CBF penumbra | 0.265 | 0.058 | 0.6948 |
| Mean FA of DWMH-FA penumbra | 0.012 | 0.923 | 0.9395 |
| Mean MD of DWMH-MD penumbra | -0.018 | 0.883 | 0.9395 |

PVWMH=periventricular white matter hyperintensity; DWMH=deep white matter hyperintensity; CBF=cerebral blood flow; FA=fractional anisotropy; MD=mean diffusivity; FDR= false discovery rate.
